# Supplementary material for: Identification and Characterization of MmuPV1 Causing Papillomatosis Outbreak in an Animal Research Facility
Source: Viruses. 2025 Sep 1;17(9):1204. doi: 10.3390/v17091204 (PMC12474283; doi:10.3390/v17091204)
Supplement: Supplementary file 1 [file viruses-17-01204-s001.zip › viruses-3815399-supplementary.pdf]

```
CLUSTAL O(1.2.4) multiple sequence alignment

HQ625439        ATGGAGATCGGCAAAGGCTACACTCTCGAGGAGGTGCTTAGATATTCTAACAAAGATGTC        60
NC_014326       ATGGAAATCGGCAAAGGCTACACTCTCGAGGAGGTGCTTAGATATTCTAACAAAGATGTC        60
PX123224        ATGGAAATCGGCAAAGGCTACACTCTCGAGGAGGTGCTTAGATATTCTAACAAAGATGTC        60
PX245442        ATGGAAATCGGCAAAGGCTACACTCTCGAGGAGGTGCTTAGATATTCTAACAAAGATGTC        60
                ***** ******************************************************

HQ625439        GTGGATTTTCATTTGTCTTGTGCTTTTTGCTCTACTACTATGGATCATAACGAGAAGGCC        120
NC_014326       GTGGATTTTCATTTGTCTTGTGCTTTTTGCTCTACTACTATGGATCATAACGAGAAGGCC        120
PX123224        GTGGATTTTCATTTGTCTTGTGCTTTTTGCTCTACTACTATGGATCATAACGAGAAGGCC        120
PX245442        GTGGATTTTCATTTGTCTTGTGCTTTTTGCTCTACTACTATGGATCATAACGAGAAGGCC        120
                ************************************************************

HQ625439        AGATTCTTACATGCTAAATTGAAATGTGTTGTTAGAGATTTTGCTTTTAAAGGTGCTTGT        180
NC_014326       AGATTCATACAGGCTAAATTGAAATGTGTTGTTAGAGATTTTGCTTTTAAAGGTGCTTGT        180
PX123224        AGATTCATACAGGCTAAATTGAAATGTGTTGTTAGAGATTTTGCTTTTAAAGGTGCTTGT        180
PX245442        AGATTCATACAGGCTAAATTGAAATGTGTTGTTAGAGATTTTGCTTTTAAAGGTGCTTGT        180
                ****** **** ************************************************

HQ625439        ATTGTGTGCCGCAGACAGCTTGCTTGCAAGGAAAAGCTTTTGCATACTAGAGTTACAGGG        240
NC_014326       ATTGTGTGCCGCAGACAGCTTGCTTGCAAGGAAAAGCTTTTGCATACTAGAGTTACAGGG        240
PX123224        ATTGTGTGCCGCAGACAGCTTGCTTGCAAGGAAAAGCTTTTGCATACTAGAGTTACAGGG        240
PX245442        ATTGTGTGCCGCAGACAGCTTGCTTGCAAGGAAAAGCTTTTGCATACTAGAGTTACAGGG        240
                ************************************************************

HQ625439        GAGGCTGATTTGGTAGAGTGCATGGCTGGTAAGAATATTGTGTTTGTTACTGTAAGATGT        300
NC_014326       GAGGCTGATTTGGTAGAGTGCATGGCTGGCAAGAATATTGTGTTTGTTACTGTAAGATGT        300
PX123224        GAGGCTGATTTGGTAGAGTGCATGGCTGGCAAGAATATTGTGTTTGTTACTGTAAGATGT        300
PX245442        GAGGCTGATTTGGTAGAGTGCATGGCTGGCAAGAATATTGTGTTTGTTACTGTAAGATGT        300
                ***************************** ******************************

HQ625439        GTTACGTGCCTGGCACTCCTTACTGCCTCTGAAAAGCTTGATGCCAAAGCGTGCGGCTTG        360
NC_014326       GTTACGTGCCTGGCACTCCTTACTGCCTCTGAAAAGCTTGATGCCAAAGCGTGCGGCTTG        360
PX123224        GTTACGTGCCTGGCACTCCTTACTGCCTCTGAAAAGCTTGATGCCAAAGCGTGCGGCTTG        360
PX245442        GTTACGTGCCTGGCACTCCTTACTGCCTCTGAAAAGCTTGATGCCAAAGCGTGCGGCTTG        360
                ************************************************************

HQ625439        CCATTTCACTTGGTGCGCCACATGTGGAGAGGCTACTGCGGGTTCTGCAAACCATCACTA        420
NC_014326       CCATTTCACTTGGTGCGCCACATGTGGAGAGGCTACTGCGGGTTCTGCAAACCATTACTA        420
PX123224        CCATTTCACTTGGTGCGCCACATGTGGAGAGGCTACTGCGGGTTCTGCAAACCATTACTA        420
PX245442        CCATTTCACTTGGTGCGCCACATGTGGAGAGGCTACTGCGGGTTCTGCAAACCATTACTA        420
                ******************************************************* ****

HQ625439        TAATGCAGGGCCCATTACCAACAATTGCTGACATCGAGATTCAGAATCTCGACTCACTTT        480
NC_014326       TAATGCAGGGCCCATTACCAACAATTGCTGACATCGAGATTCAGAATCTCGACTCACTTT        480
PX123224        TAATGCAGGGCCCATTACCAACAATTGCTGACATCGAGATTCAGAATCTCGACTCACTTT        480
PX245442        TAATGCAGGGCCCATTACCAACAATTGCTGACATCGAGATTCAGAATCTCGACTCACTTT        480
                ************************************************************

HQ625439        TGGGTGTTGGTGAGCCTGACCCACCCGATGTTGGGTCATCATCGTTGTCACCAGACTCGT        540
NC_014326       TGGGTGTTGGTGAGCCTGACCTACCCGATGTTGGGTCATCATCGTTGTCACCAGACTCGT        540
PX123224        TGGGTGTTGGTGAGCCTGACCTACCCGATGTTGGGTCATCATCGTTGTCACCAGACTCGT        540
PX245442        TGGGTGTTGGTGAGCCTGACCTACCCGATGTTGGGTCATCATCGTTGTCACCAGACTCGT        540
                ********************* **************************************

HQ625439        TAGGAGAAGAGGAGGAGCTGGAGCTGGAGACTATCGATGTAGATCCTTACAGGATTAAAA        600
NC_014326       TAGGAGAAGAGGAGGAGCTGGAGCTGGAGACTATCGATGTAGATCCTTACAGGATTAAAA        600
PX123224        TAGGAGAAGAGGAGGAGCTGGAGCTGGAGACTATCGATGTAGATCCTTACAGGATTAAAA        600
PX245442        TAGGAGAAGAGGAGGAGCTGGAGCTGGAGACTATCGATGTAGATCCTTACAGGATTAAAA        600
                ************************************************************
```

```
HQ625439        CAACCTGCTTTTGCTGCGACACTGTTCTCCGGTTCATAATTGTGACCGGAGACGACTCGG        660
NC_014326       CAACCTGCTTTTGCTGCGACACTGTTCTCCGGTTCATAATTGTGACCGGAGACGACTCGG        660
PX123224        CAACCTGCTTTTGCTGCGACACTGTTCTCCGGTTCATAATTGTGACCGGAGACGACTCGG        660
PX245442        CAACCTGCTTTTGCTGCGACACTGTTCTCCGGTTCATAATTGTGACCGGAGACGACTCGG        660
                ************************************************************

HQ625439        TGAAGGCATTCGAGTCACTGCTTCTGCAGGATCTTAGCTTTCTCTGCCCACACTGCGTCG        720
NC_014326       TGAAAGCATTCGAGTCACTGCTTCTGCAGGATCTTAGCTTTGTCTGCCCGCACTGCGTCG        720
PX123224        TGAAAGCATTCGAGTCACTGCTTCTGCAGGATCTTAGCTTTGTCTGCCCGCACTGCGTCG        720
PX245442        TGAAAGCATTCGAGTCACTGCTTCTGCAGGATCTTAGCTTTGTCTGCCCGCACTGCGTCG        720
                **** ************************************ ******* **********

HQ625439        CGTCGTACGTGAACCTCAGAAATGGAAAACGATAAGGGTACAGGGCAGTATTCTGGATGG        780
NC_014326       CGTCGTACGTGAACCTCAGAAATGGAAAACGATAAAGGTACAGGGCAGTATTCTGGATGG        780
PX123224        CGTCGTACGTGAACCTCAGAAATGGAAAACGATAAAGGTACAGGGCAGTATTCTGGATGG        780
PX245442        CGTCGTACGTGAACCTCAGAAATGGAAAACGATAAAGGTACAGGGCAGTATTCTGGATGG        780
                *********************************** ************************

HQ625439        TGTTTTATAGATAATGAGGCTGAATGTGTGGATGATGTGGGTTCCCTGGATAACTTAGAG        840
NC_014326       TGTTTTATAGATAATGAGGCTGAATGTGTGGATGATGTGGGTTCCTTGGATAACTTAGAG        840
PX123224        TGTTTTATAGATAATGAGGCTGAATGTGTGGATGATGTGGGTTCCTTGGATAACTTAGAG        840
PX245442        TGTTTTATAGATAATGAGGCTGAATGTGTGGATGATGTGGGTTCCTTGGATAACTTAGAG        840
                ********************************************* **************

HQ625439        GCATTGTTTGAGCAGAGTACCCAGGGATCATTCATTGACAGTGATGAGGTGGATCAGGGA        900
NC_014326       GCATTGTTTGAGCAGAGTACCCAGGGATCATTCATTGACAATGATGAGGTGGATCAGGGA        900
PX123224        GCATTGTTTGAGCAGAGTACCCAGGGATCATTCATTGACAATGATGAGGTGGATCAGGGA        900
PX245442        GCATTGTTTGAGCAGAGTACCCAGGGATCATTCATTGACAATGATGAGGTGGATCAGGGA        900
                **************************************** *******************

HQ625439        AATTCCTTGGCATTGCTTTCAGAGCAGTTATTTGCAACTGATGAGCAACAGATTGCAGCC        960
NC_014326       AATTCCTTGGCATTGCTTTCAGAGCAGTTATTTGCAACTGATGAGCAACAGATTGCAGCC        960
PX123224        AATTCCTTGGCATTGCTTTCAGAGCAGTTATTTGCAACTGATGAGCAACAGATTGCAGCC        960
PX245442        AATTCCTTGGCATTGCTTTCAGAGCAGTTATTTGCAACTGATGAGCAACAGATTGCAGCC        960
                ************************************************************

HQ625439        CTAAAACGAAAGTATGCCGCGACACCTAAGAAAAAAACGGTAGAAATCGAAAATCTGAGT        1020
NC_014326       CTAAAACGAAAGTATGCCGCGACACCTAAGAAAAAAACGGTAGAAATCGAAAATCTGAGT        1020
PX123224        CTAAAACGAAAGTATGCCGCGACACCTAAGAAAAAAACGGTAGAAATCGAAAATCTGAGT        1020
PX245442        CTAAAACGAAAGTATGCCGCGACACCTAAGAAAAAAACGGTAGAAATCGAAAATCTGAGT        1020
                ************************************************************

HQ625439        CCTAGACTAGAGTCCGTCAGCATTTCACCTAAAGGAAAGAGCAGGAGACGGTTGTTTGAC        1080
NC_014326       CCTAGATTAGAGTCCGTCAGCATTTCACCTAAAGGAAAGAGCAGGAGACGGTTGTTTGAC        1080
PX123224        CCTAGATTAGAGTCCGTCAGCATTTCACCTAAAGGAAAGAGCAGGAGACGGTTGTTTGAC        1080
PX245442        CCTAGATTAGAGTCCGTCAGCATTTCACCTAAAGGAAAGAGCAGGAGACGGTTGTTTGAC        1080
                ****** *****************************************************

HQ625439        AGCGGAATAGGACATGAAACTCAAGATACTCCTTCGGGGAGCGAGGTACCTATGAGCATA        1140
NC_014326       AGCGGAATAGGACATGAAACTCAAGATACTCCTTCGGGGAGCGAGGTACCTATGAGCATA        1140
PX123224        AGCGGAATAGGACATGAAACTCAAGATACTCCTTCGGGGAGCGAGGTACCTATGAGCATA        1140
PX245442        AGCGGAATAGGACATGAAACTCAAGATACTCCTTCGGGGAGCGAGGTACCTATGAGCATA        1140
                ************************************************************

HQ625439        TCTGGGTCTAGTTCAGCCAATTCAAGCATAGGAAGCCAGTGCGAGAGCGAGCAGGTAAAT        1200
NC_014326       TCTGGGTCTAGTTCAGCCAATTCAAGCATAGGAAGCCAGTGCGAGAGCGAGCAGGTAAAT        1200
PX123224        TCTGGGTCTAGTTCAGCCAATTCAAGCATAGGAAGCCAGTGCGAGAGCGAGCAGGTAAAT        1200
PX245442        TCTGGGTCTAGTTCAGCCAATTCAAGCATAGGAAGCCAGTGCGAGAGCGAGCAGGTAAAT        1200
                ************************************************************
```

```
HQ625439       AGTAACACTTTGATTTCTTCTGAAGATTTGCTTAGAACAAGTAATAGATTGGCAGGGTGC      1260
NC_014326      AGTAACACTTTGATTTCTTCTGAAGATTTGCTTAGAACAAGTAATAGATTGGCAGGGTGC      1260
PX123224       AGTAACACTTTGATTTCTTCTGAAGATTTGCTTAGAACAAGTAATAGATTGGCAGGGTGC      1260
PX245442       AGTAACACTTTGATTTCTTCTGAAGATTTGCTTAGAACAAGTAATAGATTGGCAGGGTGC      1260
               ************************************************************

HQ625439       TATGCGAGGTTTAAGGAGGCATTTGGGTGCAGCTTCACCGATCTAACGCGTAGCTTTAAG      1320
NC_014326      TATGCGAGGTTTAAGGAGGCATTTGGGTGCAGCTTCACCGATCTAACGCGTAGCTTTAAG      1320
PX123224       TATGCGAGGTTTAAGGAGGCATTTGGGTGCAGCTTCACCGATCTAACGCGTAGCTTTAAG      1320
PX245442       TATGCGAGGTTTAAGGAGGCATTTGGGTGCAGCTTCACCGATCTAACGCGTAGCTTTAAG      1320
               ************************************************************

HQ625439       AGTGATAAGACATGTAGTCCGAATTGGGTCGTAGCTGTGTTTGGGGCTAGAGAACATTTG      1380
NC_014326      AGTGATAAGACATGTAGTCCGAATTGGGTCGTAGCTGTGTTTGGGGCTAGAGAACATTTG      1380
PX123224       AGTGATAAGACATGTAGTCCGAATTGGGTCGTAGCTGTGTTTGGGGCTAGAGAACATTTG      1380
PX245442       AGTGATAAGACATGTAGTCCGAATTGGGTCGTAGCTGTGTTTGGGGCTAGAGAACATTTG      1380
               ************************************************************

HQ625439       TTGCAGGCCTTACATGATGTGTGGAAGAACACCTATGAGTACTGCCAAGATACAACAAGT      1440
NC_014326      TTGCAGGCCTTACATGATGTGTGGAAGAACACCTATGAGTACTGCCAAGATACAACAAGT      1440
PX123224       TTGCAGGCCTTACATGATGTGTGGAAGAACACCTATGAGTACTGCCAAGATACAACAAGT      1440
PX245442       TTGCAGGCCTTACATGATGTGTGGAAGAACACCTATGAGTACTGCCAAGATACAACAAGT      1440
               ************************************************************

HQ625439       TATGCAGGGAATAGAAAGGTGAACTTGCTGCTTATGGAGCTGAAGGTAGGTAGGAGCAGA      1500
NC_014326      TATGCAGGGAATAGAAAGGTGAACTTGCTGCTTATGGAGCTGAAGGTAGGTAGGAGCAGA      1500
PX123224       TATGCAGGGAATAGAAAGGTGAACTTGCTGCTTATGGAGCTGAAGGTAGGTAGGAGCAGA      1500
PX245442       TATGCAGGGAATAGAAAGGTGAACTTGCTGCTTATGGAGCTGAAGGTAGGTAGGAGCAGA      1500
               ************************************************************

HQ625439       CTCACATTGCGGAGACAGCTTTCCGCCATGTTAGGTGTGGATGAGTTGTTAATACTCGCC      1560
NC_014326      CTCACATTGCGGAGACAGCTTTCCGCCATGTTAGGTGTGGATGAGTTGTTAATACTCGCC      1560
PX123224       CTCACATTGCGGAGACAGCTTTCCGCCATGTTAGGTGTGGATGAGTTGTTAATACTCGCC      1560
PX245442       CTCACATTGCGGAGACAGCTTTCCGCCATGTTAGGTGTGGATGAGTTGTTAATACTCGCC      1560
               ************************************************************

HQ625439       GATCCGCCGAACGAGCGGAGCACGCTCGCCGCACTTTATTTTTATAATAAGGTTTTATTT      1620
NC_014326      GATCCGCCGAACGAGCGGAGCACGCTCGCCGCACTTTATTTTTATAATAAGGTTTTATTT      1620
PX123224       GATCCGCCGAACGAGCGGAGCACGCTCGCCGCACTTTATTTTTATAATAAGGTTTTATTT      1620
PX245442       GATCCGCCGAACGAGCGGAGCACGCTCGCCGCACTTTATTTTTATAATAAGGTTTTATTT      1620
               ************************************************************

HQ625439       AAAAGTCCTTCTACCGTGTTTTACGGTAGCACCCCGCTGTGGATAGCCAGCAAGACACTA      1680
NC_014326      AAAAGTCCTTCTACCATGTTTTACGGTAGCACCCCGCTGTGGATAGCCAGCAAGACACTA      1680
PX123224       AAAAGTCCTTCTACCATGTTTTACGGTAGCACCCCGCTGTGGATAGCCAGCAAGACACTA      1680
PX245442       AAAAGTCCTTCTACCATGTTTTACGGTAGCACCCCGCTGTGGATAGCCAGCAAGACACTA      1680
               *************** ********************************************

HQ625439       CTAGAGCATGCTAGTGCAACAGCCGAGTCCTTTGATTTCAGTAGTATGGTGCAGTGGGCA      1740
NC_014326      CTAGAGCATGCTAGTGCAACAGCCGAGTCCTTTGATTTCAGTAGTATGGTGCAGTGGGCA      1740
PX123224       CTAGAGCATGCTAGTGCAACAGCCGAGTCCTTTGATTTCAGTAGTATGGTGCAGTGGGCA      1740
PX245442       CTAGAGCATGCTAGTGCAACAGCCGAGTCCTTTGATTTCAGTAGTATGGTGCAGTGGGCA      1740
               ************************************************************

HQ625439       TATGACAATAGACTAAATGAGGAGGCAGAAATAGCTTATAAATATGCCTTAGAAGCAGAC      1800
NC_014326      TATGACAATAGACTAAATGAGGAGGCAGAAATAGCTTATAAATATGCCTTAGAAGCAGAC      1800
PX123224       TATGACAATAGACTAAATGAGGAGGCAGAAATAGCTTATAAATATGCCTTAGAAGCAGAC      1800
PX245442       TATGACAATAGACTAAATGAGGAGGCAGAAATAGCTTATAAATATGCCTTAGAAGCAGAC      1800
               ************************************************************
```

```
HQ625439      AGCAATAAGAATGCCCAAGCGTGGCTTAAGACTACAAACCAGGTAAAGCATGTCCGAGAC      1860
NC_014326     AGCAATAAGAATGCCCAAGCGTGGCTTAAGACTACAAACCAGGTAAAGCATGTCCGAGAC      1860
PX123224      AGCAATAAGAATGCCCAAGCGTGGCTTAAGACTACAAACCAGGTAAAGCATGTCCGAGAC      1860
PX245442      AGCAATAAGAATGCCCAAGCGTGGCTTAAGACTACAAACCAGGTAAAGCATGTCCGAGAC      1860
              ************************************************************

HQ625439      TGCTGTGCAATGGTCAGGCTATATAACAGGCAGGAAATGAAGGAAATGACAATGGCTCAG      1920
NC_014326     TGCTGTGCAATGGTCAGGCTATATAACAGGCAGGAAATGAAGGAAATGACAATGGCTCAG      1920
PX123224      TGCTGTGCAATGGTCAGGCTATATAACAGGCAGGAAATGAAGGAAATGACAATGGCTCAG      1920
PX245442      TGCTGTGCAATGGTCAGGCTATATAACAGGCAGGAAATGAAGGAAATGACAATGGCTCAG      1920
              ************************************************************

HQ625439      TGGATACGGAAGTGCTGCGATGAGACAGAGGAAGAAGGGGACTGGAAGGTTATTGCAAAC      1980
NC_014326     TGGATACGGAAGTGCTGCGATGAGACAGAGGAAGAAGGGGACTGGAAGGTTATTGCAAAC      1980
PX123224      TGGATACGGAAGTGCTGCGATGAGACAGAGGAAGAAGGGGACTGGAAGGTTATTGCAAAC      1980
PX245442      TGGATACGGAAGTGCTGCGATGAGACAGAGGAAGAAGGGGACTGGAAGGTTATTGCAAAC      1980
              ************************************************************

HQ625439      TTCCTTAGATACCAGGAAGTCAACCTCATACTGCTGCTTACAGCACTTAGGCATATGTTT      2040
NC_014326     TTCCTTAGATACCAGGAAGTCAACCTCATACTGCTGCTTACAGCACTTAGGCATATGTTT      2040
PX123224      TTCCTTAGATACCAGGAAGTCAACCTCATACTGCTGCTTACAGCACTTAGGCATATGTTT      2040
PX245442      TTCCTTAGATACCAGGAAGTCAACCTCATACTGCTGCTTACAGCACTTAGGCATATGTTT      2040
              ************************************************************

HQ625439      AAGGGTACTCCTAAAAAACACTGCCTCGTTATCACAGGTCCCCCAGATACTGGGAAGTCA      2100
NC_014326     AAGGGTACTCCTAAAAAACACTGCCTCGTTATCACAGGTCCCCCAGATACTGGGAAGTCA      2100
PX123224      AAGGGTACTCCTAAAAAACACTGCCTCGTTATCACAGGTCCCCCAGATACTGGGAAGTCA      2100
PX245442      AAGGGTACTCCTAAAAAACACTGCCTCGTTATCACAGGTCCCCCAGATACTGGGAAGTCA      2100
              ************************************************************

HQ625439      TATTTCTGTAATAGTCTGAATGGGTTTCTTAAAGGTCGTGTAATTTCATTTATGAACAGT      2160
NC_014326     TATTTCTGTAATAGTCTGAATGGGTTTCTTAAAGGTCGTGTAATTTCATTTATGAACAGT      2160
PX123224      TATTTCTGTAATAGTCTGAATGGGTTTCTTAAAGGTCGTGTAATTTCATTTATGAACAGT      2160
PX245442      TATTTCTGTAATAGTCTGAATGGGTTTCTTAAAGGTCGTGTAATTTCATTTATGAACAGT      2160
              ************************************************************

HQ625439      AGGAGTCAGTTCTGGCTGCAGCCTTTAGCAGATGCAAAAATGGGGTTCCTAGATGATGCT      2220
NC_014326     AGGAGTCAGTTCTGGCTGCAGCCTTTAGCAGATGCAAAAATGGGGTTCCTAGATGATGCT      2220
PX123224      AGGAGTCAGTTCTGGCTGCAGCCTTTAGCAGATGCAAAAATGGGGTTCCTAGATGATGCT      2220
PX245442      AGGAGTCAGTTCTGGCTGCAGCCTTTAGCAGATGCAAAAATGGGGTTCCTAGATGATGCT      2220
              ************************************************************

HQ625439      ACAACCGCTTGCTGGAACTTTGTGGATGTATATATGCGGAATGCATTAGATGGCAATCCC      2280
NC_014326     ACAACCGCTTGCTGGAACTTTATGGATGTATATATGCGGAATGCATTAGATGGCAATCCC      2280
PX123224      ACAACCGCTTGCTGGAACTTTATGGATGTATATATGCGGAATGCATTAGATGGCAATCCC      2280
PX245442      ACAACCGCTTGCTGGAACTTTATGGATGTATACATGCGGAATGCATTAGATGGCAATCCC      2280
              ********************* ********** ***************************

HQ625439      ATGCAGCTTGACATTAAGCATAGAGCACCTTTGCAGCTTAAGCTACCTCCGCTACTAATT      2340
NC_014326     ATGCAGCTTGACATTAAGCATAGAGCACCTTTGCAGCTTAAGCTACCTCCGCTACTAATT      2340
PX123224      ATGCAGCTTGACATTAAGCATAGAGCACCTTTGCAGCTTAAGCTACCTCCGCTACTAATT      2340
PX245442      ATGCAGCTTGACATTAAGCATAGAGCACCTTTGCAGCTTAAGCTACCTCCGCTACTAATT      2340
              ************************************************************

HQ625439      ACCTCAAATGTAGATGTCATGAATAATGACAATTTCAGATATCTACATAGCAGGTTGCAG      2400
NC_014326     ACCTCAAATGTAGATGTCATGAATAATGACAATTTCAGATATCTACATAGCAGGTTGCAG      2400
PX123224      ACCTCAAATGTAGATGTCATGAATAATGACAATTTCAGATATCTACATAGCAGGTTGCAG      2400
PX245442      ACCTCAAATGTAGATGTCATGAATAATGACAATTTCAGATATCTACATAGCAGGTTGCAG      2400
              ************************************************************
```

```
HQ625439       GCCTTTGAGTTTCATAAGCCTATGCCTTTAACAGCTAACGGGCAGCCAGTATATCCCCTT       2460
NC_014326      GCCTTTGAGTTTCATAAGCCTATGCCTTTAACAGCTAATGGGCAGCCAGTATATCCCCTT       2460
PX123224       GCCTTTGAGTTTCATAAGCCTATGCCTTTAACAGCTAATGGGCAGCCAGTATATCCCCTT       2460
PX245442       GCCTTTGAGTTTCATAAGCCTATGCCTTTAACAGCTAATGGGCAGCCAGTATATCCCCTT       2460
               ************************************** *********************

HQ625439       ACTAAAGCTAATTGGAAATCTTTTTTTACAAGGCTGGCTAATCAATTAGGAATCGAAGAG       2520
NC_014326      ACTAAAGCTAATTGGAAATCTTTTTTTACAAGGCTGGCTAATCAATTAGGAATCGAAGAG       2520
PX123224       ACTAAAGCTAATTGGAAATCTTTTTTTACAAGGCTGGCTAATCAATTAGGAATCGAAGAG       2520
PX245442       ACTAAAGCTAATTGGAAATCTTTTTTTACAAGGCTGGCTAATCAATTAGGAATCGAAGAG       2520
               ************************************************************

HQ625439       GAGGAGGGCGAGAATGAACAGCCTGGAAACACGTTTCGATGCAGTGCAAGACCAGATACT       2580
NC_014326      GAGGAGGGCGAGAATGAACAGCCTGGAAACACGTTTCGATGCAGTGCAAGACCAGATACT       2580
PX123224       GAGGAGGGCGAGAATGAACAGCCTGGAAACACGTTTCGATGCAGTGCAAGACCAGATACT       2580
PX245442       GAGGAGGGCGAGAATGAACAGCCTGGAAACACGTTTCGATGCAGTGCAAGACCAGATACT       2580
               ************************************************************

HQ625439       GAACCTTTACGAGAAAGGCAGTAAATGTTTAGCGGACCACATACTATATTGGGAGCTTGT       2640
NC_014326      GAACCTTTACGAGAAAGGCAGTAAATGTTTAGCGGACCACATACTATATTGGGAGCTTGT       2640
PX123224       GAACCTTTACGAGAAAGGCAGTAAATGTTTAGCGGACCACATACTATATTGGGAGCTTGT       2640
PX245442       GAACCTTTACGAGAAAGGCAGTAAATGTTTAGCGGACCACATACTATATTGGGAGCTTGT       2640
               ************************************************************

HQ625439       TAGGAAAGAAGGAGCATTGCAATTCTGTGCTCGTAGAGGGGGACTCAACAAGCTCGGACT       2700
NC_014326      TAGGAAAGAAGGAGCATTGCAATTCTGTGCTCGTAGAGGGGGACTCAACAAGCTCGGACT       2700
PX123224       TAGGAAAGAAGGAGCATTGCAATTCTGTGCTCGTAGAGGGGGACTCAACAAGCTCGGACT       2700
PX245442       TAGGAAAGAAGGAGCATTGCAATTCTGTGCTCGTAGAGGGGGACTCAACAAGCTCGGACT       2700
               ************************************************************

HQ625439       GCAACCCCTACCCAGCACCATAGGAGCCGAGAACAAGGCCAAAAGGGCAATTCAGATGCA       2760
NC_014326      GCAACCCCTACCCAGCACCATAGGAGCTGAGAACAAGGCCAAAAGGGCAATTCAGATGCA       2760
PX123224       GCAACCCCTACCCAGCACCATAGGAGCTGAGAACAAGGCCAAAAGGGCAATTCAGATGCA       2760
PX245442       GCAACCCCTACCCAGCACCATAGGAGCTGAGAACAAGGCCAAAAGGGCAATTCAGATGCA       2760
               *************************** ********************************

HQ625439       ATTGGTGCTAACATCTCTCAATGAATCACCCTTTGGTTCCGAGGAGTGGACAATGGCTGA       2820
NC_014326      ATTGGTGCTAACATCTCTCAATGAATCACCCTTTGGCTCCGAGGAGTGGACAATGGCTGA       2820
PX123224       ATTGGTGCTAACATCTCTCAATGAATCACCCTTTGGCTCCGAGGAGTGGACAATGGCTGA       2820
PX245442       ATTGGTGCTAACATCTCTCAATGAATCACCCTTTGGCTCCGAGGAGTGGACAATGGCTGA       2820
               ************************************* **********************

HQ625439       AACTAGCCGTGAGATGTATGACAGCACTGAGCCGTATGGGACTTTTAAAAAAAGCGGCGA       2880
NC_014326      AACTAGCCGTGAGATGTATGACAGCACTGAGCCGTATGGGACTTTTAAAAAAAGTGGCGA       2880
PX123224       AACTAGCCGTGAGATGTATGACAGCACTGAGCCGTATGGGACTTTTAAAAAAAGTGGCGA       2880
PX245442       AACTAGCCGTGAGATGTATGACAGCACTGAGCCGTATGGGACTTTTAAAAAAAGTGGCGA       2880
               ****************************************************** *****

HQ625439       GGAGGTGGAAGTCTATTATGGAGGAGATGAAGATAATAATGTGTCTTATATGCTCTGGAA       2940
NC_014326      GGAGGTGGAAGTCTATTATGGAGGAGATGAAGATAATAATGTGTCTTATATGCTCTGGAA       2940
PX123224       GGAGGTGGAAGTCTATTATGGAGGAGATGAAGATAATAATGTGTCTTATATGCTCTGGAA       2940
PX245442       GGAGGTGGAAGTCTATTATGGAGGAGATGAAGATAATAATGTGTCTTATATGCTCTGGAA       2940
               ************************************************************

HQ625439       GTATGTCTATGCCCAGGATGAGAACGGCAACTGGCATAAGTATCAGAGCGATTGTGACTA       3000
NC_014326      GTATGTCTATGCCCAGGATGAGAACGGCAACTGGCATAAGTATCAGAGCGATTGTGACTA       3000
PX123224       GTATGTCTATGCCCAGGATGAGAACGGCAACTGGCATAAGTATCAGAGCGATTGTGACTA       3000
PX245442       GTATGTCTATGCCCAGGATGAGAACGGCAACTGGCATAAGTATCAGAGCGATTGTGACTA       3000
               ************************************************************
```

```
HQ625439       TTATGGTGTACATTACACTGACCACAGTGGGACCCGTATCTATTATCATGATTTTGACAG      3060
NC_014326      TTATGGTGTACATTACACTGACCACAGTGGGACCCGTATCTATTATCATGATTTTGACAG      3060
PX123224       TTATGGTGTACATTACACTGACCACAGTGGGACCCGTATCTATTATCATGATTTTGACAG      3060
PX245442       TTATGGTGTACATTACACTGACCACAGTGGGACCCGTATCTATTATCATGATTTTGACAG      3060
               ************************************************************

HQ625439       TGATTCTCGCAGATATGGGGATTATTCTCACTGGACTGTGAATTATAAACACAAAACTTT      3120
NC_014326      TGATTCTCGCAGATATGGGGATTATTCTCACTGGACTGTGAATTATAAACACAAAACTTT      3120
PX123224       TGATTCTCGCAGATATGGGGATTATTCTCACTGGACTGTGAATTATAAACACAAAACTTT      3120
PX245442       TGATTCTCGCAGATATGGGGATTATTCTCACTGGACTGTGAATTATAAACACAAAACTTT      3120
               ************************************************************

HQ625439       TGAATCTTCTCCTGATAGCTCCTCCTCAGCCAAAGAAGGGCATCAAAAAACAACCAGACG      3180
NC_014326      TGAATCTTCTCCTGATAGCTCCTCCTCAGCCAAAGAAGGGCATCAAAAAACAACCAGACG      3180
PX123224       TGAATCTTCTCCTGATAGCTCCTCCTCAGCCAAAGAAGGGCATCAAAAAACAACCAGACG      3180
PX245442       TGAATCTTCTCCTGATAGCTCCTCCTCAGCCAAAGAAGGGCATCAAAAAACAACCAGACG      3180
               ************************************************************

HQ625439       GCCCGAAGACAACACCGCCACGAAGAGAACTCTTCCCACCGACACCACTGACACAGCCGC      3240
NC_014326      GCCCGAAGACAACACCGCCACGAAGAGAACTCTTCCCACCGACACCACTGACACAGCCGC      3240
PX123224       GCCCGAAGACAACACCGCCACGAAGAGAACTCTTCCCACCGACACCACTGACACAGCCGC      3240
PX245442       GCCCGAAGACAACACCGCCACGAAGAGAACTCTTCCCACCGACACCACTGACACAGCCGC      3240
               ************************************************************

HQ625439       CCCAGCCGGAGACACCATTTGGGGACGAGGCGGAGGATACGGACGACTCGGACAAGGAGA      3300
NC_014326      CCCCGCCGGAGACACCATTTGGGGACGAGGCGGAGGA---GTACGACTCGGACAAGGAGA      3297
PX123224       CCCCGCCGGAGACACCATTTGGGGACGAGGCGGAGGA---GTACGACTCGGACAAGGAGA      3297
PX245442       CCCCGCCGGAGACACCATTTGGGGACGAGGCGGAGGA---GTACGACTCGGACAAGGAGA      3297
               *** *********************************   * ******************

HQ625439       ACGACAAACCTGCATCCGGAAAGCTTGGTCAAGCGCTGCAGAGACTCCAGCGGGACCTGA      3360
NC_014326      ACGACAAACCTGCATCCGGAAAGCTTGGTCAAGCGCTGCAGAGACTCCAGCAGGACCTGA      3357
PX123224       ACGACAAACCTGCATCCGGAAAGCTTGGTCAAGCGCTGCAGAGACTCCAGCAGGACCTGA      3357
PX245442       ACGACAAACCTGCATCCGGAAAGCTTGGTCAAGCGCTGCAGAGACTCCAGCAGGACCTGA      3357
               *************************************************** ********

HQ625439       AGGATCTGCAGGACCTTGTCAACCAAACAGCATCCGGCATCACCATACTCATAGGCCAAT      3420
NC_014326      GGGATCTGCAGGACCTTGTCAACCAAACAACAGCCGGCATCACCATACTCATAGGCCAAT      3417
PX123224       GGGATCTGCAGGACCTTGTCAACCAAACAACAGCCGGCATCACCATACTCATAGGCCAAT      3417
PX245442       GGGATCTGCAGGACCTTGTCAACCAAACAACAGCCGGCATCACCATACTCATAGGCCAAT      3417
                **************************** ** ***************************

HQ625439       AATCTCTGTCAAAGGTCCGACTAACTCTTTAAAATGCTGGCGGAATAGGTTGCGTCGGAG      3480
NC_014326      AATCTCTGTCAAAGGTCCGACTAACTCTTTAAAATGCTGGCGGAATAGGTTGCGTCGGAG      3477
PX123224       AATCTCTGTCAAAGGTCCGACTAACTCTTTAAAATGCTGGCGGAATAGGTTGCGTCGGAG      3477
PX245442       AATCTCTGTCAAAGGTCCGACTAACTCTTTAAAATGCTGGCGGAATAGGTTGCGTCGGAG      3477
               ************************************************************

HQ625439       AACATATAAGCCATATAGCCGTGTATCTACTGCCTTTCAGTGGGTTGAGGATAGGGCGGA      3540
NC_014326      AACATATAAGCCATATAGCCGTGTATCTACTGCCTTTCAGTGGGTTGAGGACAGGGCGGA      3537
PX123224       AACATATAAGCCATATAGCCGTGTATCTACTGCCTTTCAGTGGGTTGAGGACAGGGCGGA      3537
PX245442       AACATATAAGCCATATAGCCGTGTATCTACTGCCTTTCAGTGGGTTGAGGACAGGGCGGA      3537
               *************************************************** ********

HQ625439       CGGGGTAGAGGTGGGGGATAGGTGGCAGGTTAGCTTTAGCAATGTACTTGTAGCTTTTGC      3600
NC_014326      CGGGGTAGAGGTGGGGGATAGGTGGCAGGTTAGCTTTAGCAATGTACTTGTAGCTTTTGC      3597
PX123224       CGGGGTAGAGGTGGGGGATAGGTGGCAGGTTAGCTTTAGCAATGTACTTGTAGCTTTTGC      3597
PX245442       CGGGGTAGAGGTGGGGGATAGGTGGCAGGTTAGCTTTAGCAATGTACTTGTAGCTTTTGC      3597
               ************************************************************
```

```
HQ625439      AGACACGCATCAAAAAGAAGTGTTTCTAAAGACTGTGACACTGCCCAAGGGCTGCTCATA      3660
NC_014326     AGACACGTATCAAAAAGAAGTGTTTCTAAAGACTGTGACACTGCCCAAGGGCTGCTCATA      3657
PX123224      AGACACGTATCAAAAAGAAGTGTTTCTAAAGACTGTGACACTGCCCAAGGGCTGCTCATA      3657
PX245442      AGACACGTATCAAAAAGAAGTGTTTCTAAAGACTGTGACACTGCCCAAGGGCTGCTCATA      3657
              ******* ****************************************************

HQ625439      CACCAGTGGCTTCTTAGACGGACTCTGATAGTGGACTCTATACACCATCCAGAATTACTG      3720
NC_014326     CACCAGTGGCTTCTTAGACGGACTCTGATAGTGGATTCTATACACCATCCAGAATTACTG      3717
PX123224      CACCAGTGGCTTCTTAGACGGACTCTGATAGTGGATTCTATACACCATCCAGAATTACTG      3717
PX245442      CACCAGTGGCTTCTTAGACGGACTCTGATAGTGGATTCTATACACCATCCAGAATTACTG      3717
              *********************************** ************************

HQ625439      TACCTGTTAGATTATTTTTGTACCATTATGGTGTCTGCTGACAGAAGCAGGCGCGTCAAG      3780
NC_014326     TACCTGTTAGATTATTTTTGTACCATTATGGTGTCTGCTGACAGAAGCAGGCGCGTCAAG      3777
PX123224      TACCTGTTAGATTATTTTTGTACCATTATGGTGTCTGCTGACAGAAGCAGGCGCGTCAAG      3777
PX245442      TACCTGTTAGATTATTTTTGTACCATTATGGTGTCTGCTGACAGAAGCAGGCGCGTCAAG      3777
              ************************************************************

HQ625439      AGGGACTCTGCGTCAAACCTATACAGACAATGTCAAGTAACCGGGAATTGTCCACCTGAT      3840
NC_014326     AGGGACTCTGCGTCAAACCTATACAGACAATGTCAAGTAACCGGGAATTGTCCACCTGAT      3837
PX123224      AGGGACTCTGCGTCAAACCTATACAGACAATGTCAAGTAACCGGGAATTGTCCACCTGAT      3837
PX245442      AGGGACTCTGCGTCAAACCTATACAGACAATGTCAAGTAACCGGGAATTGTCCACCTGAT      3837
              ************************************************************

HQ625439      GTAGTCAATAAAGTCGAAGGAAACACACTTGCTGACAGGATTCTTAAAGTTATTAGTAGC      3900
NC_014326     GTAGTCAATAAAGTCGAAGGAAACACACTTGCTGACAGGATTCTTAAAGTTATTAGTAGC      3897
PX123224      GTAGTCAATAAAGTCGAAGGAAACACACTTGCTGACAGGATTCTTAAAGTTATTAGTAGC      3897
PX245442      GTAGTCAATAAAGTCGAAGGAAACACACTTGCTGACAGGATTCTTAAAGTTATTAGTAGC      3897
              ************************************************************

HQ625439      ATTGTATACTTGGGGGGGCTTGGCATTGGAACTGGCAGAGGCTCCGGGGGCACCACTGGC      3960
NC_014326     ATTGTATACTTGGGGGGGCTGGGCATTGGAACTGGCAGAGGCTCCGGGGGCACCACTGGC      3957
PX123224      ATTGTATACTTGGGGGGGCTGGGCATTGGAACTGGCAGAGGCTCCGGGGGCACCACTGGC      3957
PX245442      ATTGTATACTTGGGGGGGCTGGGCATTGGAACTGGCAGAGGCTCCGGGGGCACCACTGGC      3957
              ******************** ***************************************

HQ625439      TATGGGCCCATAAACTCTGCTGGTGGAAGGGTAACAGGCACAGGCACGGTCATGAGGCCT      4020
NC_014326     TATGGGCCCATAAACTCTGCTGGTGGAAGGGTAACAGGCACAGGCACGGTCATGAGGCCT      4017
PX123224      TATGGGCCCATAAACTCTGCTGGTGGAAGGGTAACAGGCACAGGCACGGTCATGAGGCCT      4017
PX245442      TATGGGCCCATAAACTCTGCTGGTGGAAGGGTAACAGGCACAGGCACGGTCATGAGGCCT      4017
              ************************************************************

HQ625439      GGTGTCACTGTTGAGCCCATTGGCCCAGGGGACATAGTCACTGTAGACTCCGTGGGACCT      4080
NC_014326     GGTGTCACTGTTGAGCCCATTGGCCCAGGGGACATAGTCACTGTAGACTCTGTGGGCCCT      4077
PX123224      GGTGTCACTGTTGAGCCCATTGGCCCAGGGGACATAGTCACTGTAGACTCTGTGGGCCCT      4077
PX245442      GGTGTCACTGTTGAGCCCATTGGCCCAGGGGACATAGTCACTGTAGACTCTGTGGGCCCT      4077
              ************************************************** ***** ***

HQ625439      GGGGATTCGAGCCTTATTCCTCTACTTGAGGTGACCCCCGATGTCCCCATAAATGGGGGA      4140
NC_014326     GGAGATTCGAGCCTTATTCCTCTACTTGAGGTGACCCCCGATGTCCCTATAAATGGGGGA      4137
PX123224      GGAGATTCGAGCCTTATTCCTCTACTTGAGGTGACCCCCGATGTCCCTATAAATGGGGGA      4137
PX245442      GGAGATTCGAGCCTTATTCCTCTACTTGAGGTGACCCCCGATGTCCCTATAAATGGGGGA      4137
              ** ******************************************** ************

HQ625439      CCCGAGGTTCCTTCTAGTGGTCCAGACATAAGCACAGTGGACGTGACTTCTAGCATAGAC      4200
NC_014326     CCCGAGGTTCCTTCTAGTGGGCCAGACATAAGCACAGTGGACGTGACATCTAGCATAGAC      4197
PX123224      CCCGAGGTTCCTTCTAGTGGGCCAGACATAAGCACAGTGGACGTGACATCTAGCATAGAC      4197
PX245442      CCCGAGGTTCCTTCTAGTGGGCCAGACATAAGCACAGTGGACGTGACATCTAGCATAGAC      4197
              ******************** ************************** ************
```

```
HQ625439      CCAATATCTGACCTGTCTGTGACTGGCACCACAATCTCCAACACAGACTCTGCTGTCATT      4260
NC_014326     CCAATATCAGACCTGTCTGTGACTGGCACCACAATCTCCAACACAGACTCTGCTGTCATT      4257
PX123224      CCAATATCAGACCTGTCTGTGACTGGCACCACAATCTCCAACACAGACTCTGCTGTCATT      4257
PX245442      CCAATATCAGACCTGTCTGTGACTGGCACCACAATCTCCAACACAGACTCTGCTGTCATT      4257
              ******** ***************************************************

HQ625439      GATGTTCAGCCCTCCCCGGGTCCTCGTAGAGTCATAATCACTAGAAGTGACTTTAATAAC      4320
NC_014326     GATGTTCAGCCATCCCCGGGCCCTCGTAGAGTCATAATCACTAGAAGTGACTTTAATAAC      4317
PX123224      GATGTTCAGCCATCCCCGGGCCCTCGTAGAGTCATAATCACTAGAAGTGACTTTAATAAC      4317
PX245442      GATGTTCAGCCATCCCCGGGCCCTCGTAGAGTCATAATCACTAGAAGTGACTTTAATAAC      4317
              *********** ******** ***************************************

HQ625439      CCCTCCTACGTGTCTGTTGTGCACCCCACACAGGGGTTGGGGGAGTCTGGGGGTGTCATT      4380
NC_014326     CCCTCCTATGTGTCTGTTGTGCACCCCACACAGGGGTTGGGGGAGTCTGGGGGTGTCATT      4377
PX123224      CCCTCCTATGTGTCTGTTGTGCACCCCACACAGGGGTTGGGGGAGTCTGGGGGTGTCATT      4377
PX245442      CCCTCCTATGTGTCTGTTGTGCACCCCACACAGGGGTTGGGGGAGTCTGGGGGTGTCATT      4377
              ******** ***************************************************

HQ625439      AGTGGAGAAAGTGGAGGCATAATATCCAGCATACATGAGCTGGATAACACCACAGTCATA      4440
NC_014326     AGTGGAGAAAGTGGAGGCATAATATCCAGCATACATGAGCTGGATAACACCACAGTCATA      4437
PX123224      AGTGGAGAAAGTGGAGGCATAATATCCAGCATACATGAGCTGGATAACACCACAGTCATA      4437
PX245442      AGTGGAGAAAGTGGAGGCATAATATCCAGCATACATGAGCTGGATAACACCACAGTCATA      4437
              ************************************************************

HQ625439      GGTGCTAGGCCACCACCTGAGAGGATACTGGATGAGGTACCAGGACCCTTTGAGGACATT      4500
NC_014326     GGTGCTAGGCCACCACCTGAAAGGATATTGGATGAGGTACCAGGACCCTTTGAGGACATT      4497
PX123224      GGTGCTAGGCCACCACCTGAAAGGATATTGGATGAGGTACCAGGACCCTTTGAGGACATT      4497
PX245442      GGTGCTAGGCCACCACCTGAAAGGATATTGGATGAGGTACCAGGACCCTTTGAGGACATT      4497
              ******************** ****** ********************************

HQ625439      GAGCTTGACACATTTGTTGAGTCTAGTGGTCTTAGTGAGTTTGACATAGAGCAGCCCCTC      4560
NC_014326     GTGCTTGACACATTTGTTGAGTCTAGTGGTCTTAGTGAGTTTGACATAGAGCAGCCCCTC      4557
PX123224      GTGCTTGACACATTTGTTGAGTCTAGTGGTCTTAGTGAGTTTGACATAGAGCAGCCCCTC      4557
PX245442      GAGCTTGACACATTTGTTGAGTCTAGTGGTCTTAGTGAGTTTGACATAGAGCAGCCCCTC      4557
              * **********************************************************

HQ625439      ACTAGCACACCTGAAGGCCCGTTGCAAAGGGCGGCCACTAGATTCAGAGACCTGTATAAT      4620
NC_014326     ACTAGCACACCTGAAGGCCCGTTGCAAAGGGCGGCCACTAGATTCAGAGACCTGTATAAT      4617
PX123224      ACTAGCACACCTGAAGGCCCGTTGCAAAGGGCGGCCACTAGATTCAGAGACCTGTATAAT      4617
PX245442      ACTAGCACACCTGAAGGCCCGTTGCAAAGGGCGGCCACTAGATTCAGAGACCTGTATAAT      4617
              ************************************************************

HQ625439      AGGCGGGTGCAGCAGGTGCGTGTATCCAATCCAGAAGCTTTTCTAACTGGTCCCAGACAG      4680
NC_014326     AGGCGGGTGCAGCAGGTGCGTGTATCCAATCCAGAAGCTTTTCTAACTGGTCCCAGACAG      4677
PX123224      AGGCGGGTGCAGCAGGTGCGTGTATCCAATCCAGAAGCTTTTCTAACTGGTCCCAGACAG      4677
PX245442      AGGCGGGTGCAGCAGGTGCGTGTATCCAATCCAGAAGCTTTTCTAACTGGTCCCAGACAG      4677
              ************************************************************

HQ625439      GCGGTAGTATTTGAAAATCCCGCCTTTGAGCCTGGGAGCCTGGATTTTGAACTTCCCGCC      4740
NC_014326     GCGGTAGTATTTGAAAATCCCGCCTTTGAGCCTGGGAGCCTGGATTTTGAACTTCCCGCC      4737
PX123224      GCGGTAGTATTTGAAAATCCCGCCTTTGAGCCTGGGAGCCTGGATTTTGAACTTCCCGCC      4737
PX245442      GCGGTAGTATTTGAAAATCCCGCCTTTGAGCCTGGGAGCCTGGATTTTGAACTTCCCGCC      4737
              ************************************************************

HQ625439      GGTCCTCCTGTAGCTGCACCTGACCCTGAGTACACTGATGTGGTCCACCTAGGGCGTCAG      4800
NC_014326     AGTCCTCCTGTAGCTGCACCTGACCCTGAGTACACTGATGTGGTCCACCTAGGGCGTCAG      4797
PX123224      AGTCCTCCTGTAGCTGCACCTGACCCTGAGTACACTGATGTGGTCCACCTAGGGCGTCAG      4797
PX245442      AGTCCTCCTGTAGCTGCACCTGACCCTGAGTACACTGATGTGGTCCACCTAGGGCGTCAG      4797
               ***********************************************************
```

```
HQ625439        AGGTTCTCTGAGGTGAACAGAGTAATTAGAGTGAGCAGGTTGGGGCAACGTGCATCCATG        4860
NC_014326       AGGTTCTCTGAGGTGAACAGAGTAATTAGAGTGAGCAGGTTGGGGCAACGTGCATCTATG        4857
PX123224        AGGTTCTCTGAGGTGAACAGAGTAATTAGAGTGAGCAGGTTGGGGCAACGTGCATCTATG        4857
PX245442        AGGTTCTCTGAGGTGAACAGAGTAATTAGAGTGAGCAGGTTGGGGCAACGTGCATCTATG        4857
                ******************************************************** ***

HQ625439        AAGACTAGGAGTGGTCTTATAATTGGTGGGAAAGTGCACTTCTATACAGATTTATCCCCT        4920
NC_014326       AAGACTAGGAGTGGTCTTATAATTGGTGGGAAAGTGCACTTCTATACAGATTTATCCCCT        4917
PX123224        AAGACTAGGAGTGGTCTTATAATTGGTGGGAAAGTGCACTTCTATACAGATTTATCCCCT        4917
PX245442        AAGACTAGGAGTGGTCTTATAATTGGTGGGAAAGTGCACTTCTATACAGATTTATCCCCT        4917
                ************************************************************

HQ625439        GTTGCTACGGACATTGAAATGCACACATTAGGTGAGATCAGTGGTACTGAAGAGCTGATT        4980
NC_014326       GTTGCTACGGACATTGAAATGCACACATTAGGTGAGATCAGTGGTACTGAAGAGCTGATT        4977
PX123224        GTTGCTACGGACATTGAAATGCACACATTAGGTGAGATCAGTGGTACTGAAGAGCTGATT        4977
PX245442        GTTGCTACGGACATTGAAATGCACACATTAGGTGAGATCAGTGGTACTGAAGAGCTGATT        4977
                ************************************************************

HQ625439        GATGGTCTTGGAAGCTCTTCAGTAATTGAGTTCCCAAGGGGGGTTGAGTCTGTAGAGCTT        5040
NC_014326       GATGGTCTTGGAAGCTCTTCAGTAATTGAGTTCCCAAGGGGGGTTGAGTCTGTAGAGCTT        5037
PX123224        GATGGTCTTGGAAGCTCTTCAGTAATTGAGTTCCCAAGGGGGGTTGAGTCTGTAGAGCTT        5037
PX245442        GATGGTCTTGGAAGCTCTTCAGTAATTGAGTTCCCAAGGGGGGTTGAGTCTGTAGAGCTT        5037
                ************************************************************

HQ625439        CCAGATGGCTCTGACTCAGTGAATGAGCTACTTGACACCGATAGTGCTGATTTTTCTTCC        5100
NC_014326       CCAGATGGCTCTGACTCAGTGAATGAGCTACTTGACACCGATAGTGCTGATTTTTCTTCC        5097
PX123224        CCAGATGGCTCTGACTCAGTGAATGAGCTACTTGACACCGATAGTGCTGATTTTTCTTCC        5097
PX245442        CCAGATGGCTCTGACTCAGTGAATGAGCTACGTGACACCGATAGTGCTGATTTTTCTTCC        5097
                ******************************* ****************************

HQ625439        TCTAGGCTTGAACTACTTATAGGTAATGGGACAAGCCGTTTTGTGATGCCTGACTTGGTC        5160
NC_014326       TCTAGGCTTGAACTACTTATAGGTAATGGGACAAGCCGTTTTGTGATGCCTGACTTGGTC        5157
PX123224        TCTAGGCTTGAACTACTTATAGGTAATGGGACAAGCCGTTTTGTGATGCCTGACTTGGTC        5157
PX245442        TCTAGGCTTGAACTACTTATAGGTAATGGGACAAGCCGTTTTGTGATGCCTGACTTGGTC        5157
                ************************************************************

HQ625439        GAAACTCTAGGCCCAGACATGTTTTTCCCCAGTATCGACTCAGGCACGGTTATACCACAC        5220
NC_014326       GAAACTCTAGGCCCAGACATGTTTTTTCCCAGTATCGACTCAGGCACGGTTATACCACAC        5217
PX123224        GAAACTCTAGGCCCAGACATGTTTTTTCCCAGTATCGACTCAGGCACGGTTATACCACAC        5217
PX245442        GAAACTCTAGGCCCAGACATGTTTTTTCCCAGTATCGACTCAGGCACGGTTATACCACAC        5217
                ************************** *********************************

HQ625439        CCTCAGGATAATTATGTTCCTATTATTCTGCCAGCTGCGGATCTATTCCCAGCTTCTACG        5280
NC_014326       CCTCAAGATAATTATGTTCCTATTATTCTGCCAGCTGCGGATCTATTCCCAGCTTCTACT        5277
PX123224        CCTCAAGATAATTATGTTCCTATTATTCTGCCAGCTGCGGATCTATTCCCAGCTTCTACT        5277
PX245442        CCTCAAGATAATTATGTTCCTATTATTCTGCCAGCTGCGGATCTATTCCCAGCTTCTACT        5277
                ***** ***************************************************** 

HQ625439        GTTATAAGTGTGGATGATGACTTTGCTGATTTTTATTTGCACCCCAGTCTCCGTAAACGT        5340
NC_014326       GTTATAAGTGTGGATGATGACTTTGCTGATTTTTATTTGCACCCCAGTCTCCGTAAACGC        5337
PX123224        GTTATAAGTGTGGATGATGACTTTGCTGATTTTTATTTGCACCCCAGTCTCCGTAAACGC        5337
PX245442        GTTATAAGTGTGGATGATGACTTTGCTGATTTTTATTTGCACCCCAGTCTCCGTAAACGC        5337
                *********************************************************** 

HQ625439        AAACGAAAATATCGTATTTATTGATATTTTTCAGATGGCAATGTGGACACCCCAGACCGG        5400
NC_014326       AAACGAAAATATCGTATTTATTGATATTTTTCAGATGGCAATGTGGACACCCCAGACCGG        5397
PX123224        AAACGAAAATATCGTATTTATTGATATTTTTCAGATGGCAATGTGGACACCCCAGACCGG        5397
PX245442        AAACGAAAATATCGTATTTATTGATATTTTTCAGATGGCAATGTGGACACCCCAGACCGG        5397
                ************************************************************
```

```
HQ625439      GAAGCTTTACCCCCCACCTACAACTCCAGTGGCAAAAGTGCAGAGCACAGACGAATATGT      5460
NC_014326     GAAGCTTTACCTCCCACCTACAACTCCAGTGGCAAAAGTGCAGAGCACAGACGAATATGT      5457
PX123224      GAAGCTTTACCTCCCACCTACAACTCCAGTGGCAAAAGTGCAGAGCACAGACGAATATGT      5457
PX245442      GAAGCTTTACCTCCCACCTACAACTCCAGTGGCAAAAGTGCAGAGCACAGACGAATATGT      5457
              *********** ************************************************

HQ625439      GTACCCTACGTCTCTCTCCTGTCATGCACACACGGACCGTTTGCTAACAGTGGGCCACCC      5520
NC_014326     GTACCCTACGTCTCTCTTCTGTCATGCACACACGGACCGTTTGCTAACAGTGGGCCACCC      5517
PX123224      GTACCCTACGTCTCTCTTCTGTCATGCACACACGGACCGTTTGCTAACAGTGGGCCACCC      5517
PX245442      GTACCCTACGTCTCTCTTCTGTCATGCACACACGGACCGTTTGCTAACAGTGGGCCACCC      5517
              ***************** ******************************************

HQ625439      GTATTTTTCTGTCCTTGACAATGACAAGGTCACTGTGCCTAAAGTGTCTGGCAACCAATA      5580
NC_014326     TTTTTTTTCTGTCATTGACAATGACAAGGTCACTGTGCCTAAAGTGTCTGGCAACCAATA      5577
PX123224      TTTTTTTTCTGTCATTGACAATGACAAGGTCACTGTGCCTAAAGTGTCTGGCAACCAATA      5577
PX245442      TTTTTTTTCTGTCATTGACAATGACAAGGTCACTGTGCCTAAAGTGTCTGGCAACCAATA      5577
               * ********** **********************************************

HQ625439      TAGAGTTTTCAGACTTAAATTCCCAGATCCAAATAAATTTGCATTGCCCCAAAAGGATTT      5640
NC_014326     TAGGGTTTTCAGACTTAAATTCCCAGATCCAAATAAATTTGCATTGCCCCAAAAGGATTT      5637
PX123224      TAGGGTTTTCAGACTTAAATTCCCAGATCCAAATAAATTTGCATTGCCCCAAAAGGATTT      5637
PX245442      TAGGGTTTTCAGACTTAAATTCCCAGATCCAAATAAATTTGCATTGCCCCAAAAGGATTT      5637
              *** ********************************************************

HQ625439      CTATGATCCTGAGAAAGAACGGTTAGTGTGGAGGTTAAGGGGTCTGGAAATTGGAAGAGG      5700
NC_014326     CTATGATCCTGAGAAAGAACGGTTAGTGTGGAGGTTAAGGGGTCTGGAAATTGGAAGAGG      5697
PX123224      CTATGATCCTGAGAAAGAACGGTTAGTGTGGAGGTTAAGGGGTCTGGAAATTGGAAGAGG      5697
PX245442      CTATGATCCTGAGAAAGAACGGTTAGTGTGGAGGTTAAGGGGTCTGGAAATTGGAAGAGG      5697
              ************************************************************

HQ625439      TGGCCCATTAGGGATTGGCACTACCGGGCACCCCCTTTTTAACAAGCTTGGAGACACGGA      5760
NC_014326     TGGCCCATTAGGGATTGGCACTACCGGGCACCCCCTTTTTAACAAGCTTGGAGACACGGA      5757
PX123224      TGGCCCATTAGGGATTGGCACTACCGGGCACCCCCTTTTTAACAAGCTTGGAGACACGGA      5757
PX245442      TGGCCCATTAGGGATTGGCACTACCGGGCACCCCCTTTTTAACAAGCTTGGAGACACGGA      5757
              ************************************************************

HQ625439      AAATCCAAACAAATATCAGCAAGGCTCTAAGGATAATAGGCAGAACACTTCCATGGACCC      5820
NC_014326     AAATCCAAATAAATATCAGCAAGGCTCTAAGGATAATAGGCAGAACACTTCCATGGACCC      5817
PX123224      AAATCCAAATAAATATCAGCAAGGCTCTAAGGATAATAGGCAGAACACTTCCATGGACCC      5817
PX245442      AAATCCAAATAAATATCAGCAAGGCTCTAAGGATAATAGGCAGAACACTTCCATGGACCC      5817
              ********* **************************************************

HQ625439      CAAACAAACACAGCTGTTTATTGTTGGATGTGAACCCCCTACAGGGGAACACTGGGATGT      5880
NC_014326     CAAACAAACACAGCTGTTTATTGTTGGCTGTGAACCCCCTACAGGGGAACACTGGGATGT      5877
PX123224      CAAACAAACACAGCTGTTTATTGTTGGCTGTGAACCCCCTACAGGGGAACACTGGGATGT      5877
PX245442      CAAACAAACACAGCTGTTTATTGTTGGCTGTGAACCCCCTACAGGGGAACACTGGGATGT      5877
              *************************** ********************************

HQ625439      AGCTAAGCCCTGTGGAGCTCTGGAAAAGGGTGACTGCCCTCCTATCCAACTTGTAAATAG      5940
NC_014326     AGCTAAGCCCTGTGGAGCTCTGGAGAAGGGTGACTGCCCTCCTATCCAACTTGTAAATAG      5937
PX123224      AGCTAAGCCCTGTGGAGCTCTGGAGAAGGGTGACTGCCCTCCTATCCAACTTGTAAATAG      5937
PX245442      AGCTAAGCCCTGTGGAGCTCTGGAGAAGGGTGACTGCCCTCCTATCCAACTTGTAAATAG      5937
              ************************ ***********************************

HQ625439      TGTAATCGAGGATGGGGATATGTGTGACATTGGCTTTGGGAATATGAACTTCAAAGAGCT      6000
NC_014326     TGTAATTGAGGATGGGGATATGTGTGACATTGGCTTTGGGAATATGAACTTCAAAGAGCT      5997
PX123224      TGTAATTGAGGATGGGGATATGTGTGACATTGGCTTTGGGAATATGAACTTCAAAGAGCT      5997
PX245442      TGTAATTGAGGATGGGGATATGTGTGACATTGGCTTTGGGAATATGAACTTCAAAGAGCT      5997
              ****** *****************************************************
```

```
HQ625439      GCAGCAGGACAGGAGTGGTGTGCCTCTTGATATTGTATCTACCCGGTGCAAATGGCCCGA      6060
NC_014326     GCAGCAGGATAGGAGTGGTGTGCCTCTTGATATTGTATCTACCCGGTGCAAATGGCCCGA      6057
PX123224      GCAGCAGGATAGGAGTGGTGTGCCTCTTGATATTGTATCTACCCGGTGCAAATGGCCCGA      6057
PX245442      GCAGCAGGATAGGAGTGGTGTGCCTCTTGATATTGTATCTACCCGGTGCAAATGGCCCGA      6057
              ********* **************************************************

HQ625439      CTTTCTGAAAATGACCAATGAGGCATATGGGGATAAGATGTTCTTCTTTGGAAGGAGAGA      6120
NC_014326     CTTTCTGAAAATGACCAATGAGGCATATGGGGATAAGATGTTCTTCTTTGGAAGGAGAGA      6117
PX123224      CTTTCTGAAAATGACCAATGAGGCATATGGGGATAAGATGTTCTTCTTTGGAAGGAGAGA      6117
PX245442      CTTTCTGAAAATGACCAATGAGGCATATGGGGATAAGATGTTCTTCTTTGGAAGGAGAGA      6117
              ************************************************************

HQ625439      GCAAGTGTATGCAAGACACTTTTTCACCAGGAATGGCTCTGTGGGGGAGCCCATACCAAA      6180
NC_014326     GCAAGTGTATGCAAGACACTTTTTCACCAGGAATGGCTCTGTGGGGGAGCCCATACCAAA      6177
PX123224      GCAAGTGTATGCAAGACACTTTTTCACCAGGAATGGCTCTGTGGGGGAGCCCATACCAAA      6177
PX245442      GCAAGTGTATGCAAGACACTTTTTCACCAGGAATGGCTCTGTGGGGGAGCCCATACCAAA      6177
              ************************************************************

HQ625439      CTCTGTGAGTCCCAGTGACTTTTACTACGCACCTGACAGCACACAGGACCAGAAGACACT      6240
NC_014326     CTCTGTGAGTCCCAGTGACTTTTACTACGCACCCGACAGCACACAGGACCAGAAGACACT      6237
PX123224      CTCTGTGAGTCCCAGTGACTTTTACTACGCACCCGACAGCACACAGGACCAGAAGACACT      6237
PX245442      CTCTGTGAGTCCCAGTGACTTTTACTACGCACCCGACAGCACACAGGACCAGAAGACACT      6237
              ********************************* **************************

HQ625439      CGCTCCCTCCGTGTACTTTGGAACTCCTAGTGGGTCTCTTGTGTCTAGTGATGGTCAGCT      6300
NC_014326     CGCACCCTCCGTGTACTTTGGAACTCCTAGTGGGTCACTTGTGTCGAGTGATGGTCAGCT      6297
PX123224      CGCACCCTCCGTGTACTTTGGAACTCCTAGTGGGTCACTTGTGTCGAGTGATGGTCAGCT      6297
PX245442      CGCACCCTCCGTGTACTTTGGAACTCCTAGTGGGTCACTTGTGTCGAGTGATGGTCAGCT      6297
              *** ******************************** ******** **************

HQ625439      GTTTAACAGGCCATTTTGGCTTCAAAGGGCTCAGGGAAACAATAATGGTGTGTGCTGGCA      6360
NC_014326     GTTTAACAGGCCATTTTGGCTTCAAAGGGCTCAGGGAAACAATAATGGTGTGTGCTGGCA      6357
PX123224      GTTTAACAGGCCATTTTGGCTTCAAAGGGCTCAGGGAAACAATAATGGTGTGTGCTGGCA      6357
PX245442      GTTTAACAGGCCATTTTGGCTTCAAAGGGCTCAGGGAAACAATAATGGTGTGTGCTGGCA      6357
              ************************************************************

HQ625439      CAATGAGCTCTTTGTTACTGTTGTCGACAACACAAGAAATACAAACTTTACTATCTCCCA      6420
NC_014326     CAATGAGCTCTTTGTTACTGTTGTCGACAACACAAGGAATACAAACTTTACTATCTCCCA      6417
PX123224      CAATGAGCTCTTTGTTACTGTTGTCGACAACACAAGGAATACAAACTTTACTATCTCCCA      6417
PX245442      CAATGAGCTCTTTGTTACTGTTGTCGACAACACAAGGAATACAAACTTTACTATCTCCCA      6417
              ************************************ ***********************

HQ625439      GCAAACCAACACACCAAACCCAGATACATATGACTCTACTAATTTTAAGAACTATTTAAG      6480
NC_014326     GCAAACCAACACACCAAACCCAGATACATATGACTCTACTAATTTTAAAAACTATTTAAG      6477
PX123224      GCAAACCAACACACCAAACCCAGATACATATGACTCTACTAATTTTAAAAACTATTTAAG      6477
PX245442      GCAAACCAACACACCAAACCCAGATACATATGACTCTACTAATTTTAAAAACTATTTAAG      6477
              ************************************************ ***********

HQ625439      ACATGTGGAACAATTTGAGCTGTCCCTTATTGCTCAACTGTGTAAGGTTCCACTTGACCC      6540
NC_014326     ACATGTGGAACAATTTGAGCTGTCCCTTATTGCTCAACTGTGTAAGGTTCCACTTGACCC      6537
PX123224      ACATGTGGAACAATTTGAGCTGTCCCTTATTGCTCAACTGTGTAAGGTTCCACTTGACCC      6537
PX245442      ACATGTGGAACAATTTGAGCTGTCCCTTATTGCTCAACTGTGTAAGGTTCCACTTGACCC      6537
              ************************************************************

HQ625439      GGGTGTGCTTGCCCATATAAACACTATGAACCCAACCATCTTGGAGAACTGGAACTTGGG      6600
NC_014326     GGGTGTGCTTGCCCATATAAACACTATGAACCCAACCATCTTGGAGAACTGGAACTTGGG      6597
PX123224      GGGTGTGCTTGCCCATATAAACACTATGAACCCAACCATCTTGGAGAACTGGAACTTGGG      6597
PX245442      GGGTGTGCTTGCCCATATAAACACTATGAACCCAACCATCTTGGAGAACTGGAACTTGGG      6597
              ************************************************************
```

```
HQ625439      TTTTGTACCTCCCCCACAGCAGTCCATCTCTGATGACTATAGGTATATAACATCATCGGC      6660
NC_014326     TTTTGTACCTCCCCCACAGCAGTCCATCTCTGATGACTATAGGTATATAACATCATCGGC      6657
PX123224      TTTTGTACCTCCCCCACAGCAGTCCATCTCTGATGACTATAGGTATATAACATCATCGGC      6657
PX245442      TTTTGTACCTCCCCCACAGCAGTCCATCTCTGATGACTATAGGTATATAACATCATCGGC      6657
              ************************************************************

HQ625439      AACTCGCTGTCCAGATCAGAATCCGCCCAAGGAAAGAGAGGATCCTTACAAGGGTCTTAT      6720
NC_014326     AACTCGCTGTCCAGATCAGAATCCGCCCAAGGAAAGAGAGGATCCTTACAAGGGTCTTAT      6717
PX123224      AACTCGCTGTCCAGATCAGAATCCGCCCAAGGAAAGAGAGGATCCTTACAAGGGTCTTAT      6717
PX245442      AACTCGCTGTCCAGATCAGAATCCGCCCAAGGAAAGAGAGGATCCTTACAAGGGTCTTAT      6717
              ************************************************************

HQ625439      ATTTTGGGAAGTTGATCTTACTGAGAGGTTTTCTCAGGACCTTGATCAGTTTGCTCTGGG      6780
NC_014326     ATTTTGGGAAGTTGATCTTACTGAGAGGTTTTCTCAGGACCTTGATCAGTTTGCTCTGGG      6777
PX123224      ATTTTGGGAAGTTGATCTTACTGAGAGGTTTTCTCAGGACCTTGATCAGTTTGCTCTGGG      6777
PX245442      ATTTTGGGAAGTTGATCTTACTGAGAGGTTTTCTCAGGACCTTGATCAGTTTGCTCTGGG      6777
              ************************************************************

HQ625439      ACGAAAGTTTCTGTATCAAGCTGGTATACGTACTGCTGTTACGGGCCGCGGGGTCAAAAG      6840
NC_014326     ACGAAAGTTTCTGTATCAAGCTGGTATACGTACTGCTGTTACGGGCCGCGGGGTCAAAAG      6837
PX123224      ACGAAAGTTTCTGTATCAAGCTGGTATACGTACTGCTGTTACGGGCCGCGGGGTCAAAAG      6837
PX245442      ACGAAAGTTTCTGTATCAAGCTGGTATACGTACTGCTGTTACGGGCCGCGGGGTCAAAAG      6837
              ************************************************************

HQ625439      GGCAGCGTCTACAACCTCTGCGTCTTCTAGACGAGTTGTAAAAAGGAAGAGGGGAAGCAA      6900
NC_014326     GGCAGCGTCTACAACCTCTGCGTCTTCTAGACGAGTTGTAAAACGGAAGAGGGGAAGCAA      6897
PX123224      GGCAGCGTCTACAACCTCTGCGTCTTCTAGACGAGTTGTAAAACGGAAGAGGGGAAGCAA      6897
PX245442      GGCAGCGTCTACAACCTCTGCGTCTTCTAGACGAGTTGTAAAACGGAAGAGGGGAAGCAA      6897
              ******************************************* ****************

HQ625439      ATAACTGAACTGGTGCTACTAACTGAATTTGGTGCTATGACTCCGGTATTATGAAGTTCT      6960
NC_014326     ATAACTGAACTGGTGCTACTAACTGAA----------TGACTCCGGTATTATGAAGTTCT      6947
PX123224      ATAACTGAACTGGTGCTACTAACTGAA----------TGACTCCGGTATTATGAAGTTCT      6947
PX245442      ATAACTGAACTGGTGCTACTAACTGAA----------TGACTCCGGTATTATGAAGTTCT      6947
              ***************************          ***********************

HQ625439      TGTATTGTATAACTGTTTACTGGGGGCTTACTGTGTATAGGGGGCTTGAGTTGTTTGTCT      7020
NC_014326     TGTATTGTATAACTGTTTACTGGGGGCTTACTGTGTATAGGGGGCTTGAGTTGTTTGTCT      7007
PX123224      TGTATTGTATAACTGTTTACTGGGGGCTTACTGTGTATAGGGGGCTTGAGTTGTTTGTCT      7007
PX245442      TGTATTGTATAACTGTTTACTGGGGGCTTACTGTGTATAGGGGGCTTGAGTTGTTTGTCT      7007
              ************************************************************

HQ625439      GTTCTTGTCCATGTCCTTGTGATGTACTTTTGCAACTTAAATAAATGACTAATGCTGACC      7080
NC_014326     GTTCTTGTCCATGTCCTTGTGATGTACTTTTGCAACTTAAATAAATGACTAATGCTGACC      7067
PX123224      GTTCTTGTCCATGTCCTTGTGATGTACTTTTGCAACTTAAATAAATGACTAATGCTGACC      7067
PX245442      GTTCTTGTCCATGTCCTTGTGATGTACTTTTGCAACTTAAATAAATGACTAATGCTGACC      7067
              ************************************************************

HQ625439      AGTGTGCCTCGCCTCATTCTTCAGCTCGCACCTGGGCTCACTTTGTGCCAGACTGTCATA      7140
NC_014326     AGTGTGCCTCGCCTCATTCTTTAGCTCGCACCTGGGCTCACTTTGTGCCAGACTGTCATA      7127
PX123224      AGTGTGCCTCGCCTCATTCTTTAGCTCGCACCTGGGCTCACTTTGTGCCAGACTGTCATA      7127
PX245442      AGTGTGCCTCGCCTCATTCTTTAGCTCGCACCTGGGCTCACTTTGTGCCAGACTGTCATA      7127
              ********************* **************************************

HQ625439      ACAAACAGTCTCTGTTGGCTGTGTGCTCTCTAATTTCTTGAAAAGACGTGTTTTGACGAA      7200
NC_014326     ACAAACAGTCTCTGTTGGCTGTGTGCTCTCTAATTTCTCGAAAAGACGTGTTTTGACGAA      7187
PX123224      ACAAACAGTCTCTGTTGGCTGTGTGCTCTCTAATTTCTCGAAAAGACGTGTTTTGACGAA      7187
PX245442      ACAAACAGTCTCTGTTGGCTGTGTGCTCTCTAATTTCTCGAAAAGACGTGTTTTGACGAA      7187
              ************************************** *********************
```

```
HQ625439       GGACCGTTTTCGGTCGGGCGCCAGTATCAGCATAAACTCCAGCCAATTTGGCCAAGGTAA       7260
NC_014326      GGACCGTTTTCGGTCGGGCGCCAGTATCAGCATAAACTCCAGCCAATTTGGCCAAGGTAA       7247
PX123224       GGACCGTTTTCGGTCGGGCGCCAGTATCAGCATAAACTCCAGCCAATTTGGCCAAGGTAA       7247
PX245442       GGACCGTTTTCGGTCGGGCGCCAGTATCAGCATAAACTCCAGCCAATTTGGCCAAGGTAA       7247
               ************************************************************

HQ625439       GGAAATGACTAACTGTCTTGGAACAGATGCGTGTCCTGGCAATTATCCGCGTACCGTTTT       7320
NC_014326      GGAAATGACTAACTGTCTTGGAACAGATGCGTGTCCTGGCAATTATCCGCGTACCGTTTT       7307
PX123224       GGAAATGACTAACTGTCTTGGAACAGATGCGTGTCCTGGCAATTATCCGCGTACCGTTTT       7307
PX245442       GGAAATGACTAACTGTCTTGGAACAGATGCGTGTCCTGGCAATTATCCGCGTACCGTTTT       7307
               ************************************************************

HQ625439       CGGTCGGGTAAAAAAGGCGCCAAGCTAAGCATGATTCAGAGTTCCATTGTGTTCTGCCAA       7380
NC_014326      CGGTCGGGTAAAAAAGGCGCCAAGCTAAGCATGATTCAGAGTTCCATTGTGTTCTGCCAA       7367
PX123224       CGGTCGGGTAAAAAAGGCGCCAAGCTAAGCATGATTCAGAGTTCCATTGTGTTCTGCCAA       7367
PX245442       CGGTCGGGTAAAAAAGGCGCCAAGCTAAGCATGATTCAGAGTTCCATTGTGTTCTGCCAA       7367
               ************************************************************

HQ625439       GTACAGGTGTGGTGTTCTGGAACGGTCGTACAATTAATCTTTGAGCTGATGGTTGGCAAC       7440
NC_014326      GTACAGGTGTGGTGTTCTGGAACGGTCGTACAATTAATCTTTGAGCTGATGGTTGGCAAC       7427
PX123224       GTACAGGTGTGGTGTTCTGGAACGGTCGTACAATTAATCTTTGAGCTGATGGTTGGCAAC       7427
PX245442       GTACAGGTGTGGTGTTCTGGAACGGTCGTACAATTAATCTTTGAGCTGATGGTTGGCAAC       7427
               ************************************************************

HQ625439       AATTATTTCCCTCTGAAAAAATTTAGGTGGAGCGGGAACGGTCGCATATAAGTATCAGTG       7500
NC_014326      AATTATTTCCCTCTGAAAAAATTTAGGTGGAGCGGGAACGGTCGCATATAAGTATCAGTG       7487
PX123224       AATTATTTCCCTCTGAAAAAATTTAGGTGGAGCGGGAACGGTCGCATATAAGTATCAGTG       7487
PX245442       AATTATTTCCCTCTGAAAAAATTTAGGTGGAGCGGGAACGGTCGCATATAAGTATCAGTG       7487
               ************************************************************

HQ625439       TGCCCCCATGACCGTATTCGTTC       7523
NC_014326      TGCCCCCATAACCGTATTCGTTC       7510
PX123224       TGCCCCCATAACCGTATTCGTTC       7510
PX245442       TGCCCCCATAACCGTATTCGTTC       7510
               ********* *************
```
